# Annotations

**Published:** 1900-05-12

**Authors:** 


### Annotations.

### Wounds in War.

When the medical history of the war in South Africa comes to be written it will no doubt be found that many of our preconceived notions in regard to military surgery will require modification. As yet it would be premature to speak positively as to the extent to which this will be the case, but enough has already been communicated by the various surgeons at the seat of war to enable us to satisfy ourselves that considerable modifications will have to be made in not a few of the conclusions which have been generally accepted as to the character and treatment of wounds received in war. Moreover, we have learned enough to show that, although antiseptic measures, first aid dressing, and careful surgery have done much to protect our soldiers from some, at least, of the perils of war, those, namely, which, in times past, have so often rendered military hospitals even more dangerous than battlefields, antiseptics have failed in certain directions to do all that was hoped of them—failed, not in consequence of any error in the theories on which they are founded, or of any imperfection in the technique by which these theories are put in practice, but from the absolute impossibility of obtaining, under the urgent circumstances of war, some of the appliances which are necessary, and that degree of protection from septic influences which is essential to the due carrying out of the details of aseptic surgery as now practised in our great civil hospitals. The disappointment has been greatest, perhaps, in regard to abdominal surgery, while, on the other hand, it has been in abdominal injuries even more than any others that surprising results have been obtained by a line of conservatism in surgical treatment, or, rather, of non-intervention, which up to the moment of the outbreak of this war had been coming to be regarded more and more as inexcusable. Nothing has been more striking in the surgical history of the war than the good, the unexpectedly good results which have been met with in cases where no one could doubt that the abdominal cavity had been traversed by bullets which must in their course have produced no one knows how many penetrating wounds of the intestines. On the other hand, nothing has been more distressing than the small success that has followed surgical intervention in similar cases, and when one hears of the flies, the dust, the heat, amid which operations were done, and of the difficulty of procuring a sufficiency of clean water to ensure the elementary essentials of aseptic work, one sees that if surgery of the highest type is to be carried to the very front some modification of technique will have to be devised which shall set surgeons free from some of the requirements, such, for example, as that of clean water, which are so easily supplied in civil hospitals as to be taken for granted, and yet are so difficult of attainment in the very front as to have again and again stood in the way of success. There is an element of bathos in the thought of skilled surgeons, with every appliance at their fingers' ends that science can devise, being barred from success by the lack of a little clean water. Yet such seems at times to have been the case, and if one were to try to peep into the future one would hope that the next advance in aseptic surgery will be to do without water. This is a thing the possibility of which has long been known, and although it may not be the best method where one has one's choice, a practical method of antisepsis without water would more than anything else render high class surgery possible on the very field. The next point which seems to have been practically demonstrated is that, although a very large proportion of the wounded can be properly removed from the field of battle, and although after a drawn battle, where the ground may next day be traversed again by contending forces, removal of the wounded is a necessity at whatever risk, still, to a certain number of the severely wounded, especially those whose wounds involve the large bones, such as the femur or the pelvis, this risk is so great that removal may to them mean death. The teaching seems to be that where it is possible, and especially when an army is advancing, those who are severely wounded should be left where they are and treated on the field. Over a rough country it is easier to carry a tent than a man, especially a wounded one, and it is quite possible that in the future some system may be devised for treating, during the first few days at least, actually where they fall, those whose injuries are such as to be greatly aggravated by removal.

### The Profession and the Midwives.

At the conference on medical organisation which met in Manchester on May 1st for the discussion of some of the many questions which are agitating the medical world, various topics came under consideration, one of which was, of course, the Midwives Bill, in regard to which a resolution was passed protesting against any legislation which will have the effect of giving a legal status to midwives as a class. That a conference of intelligent beings, as Mr. Horsley somewhat sarcastically called them, could pass such a resolution at the present time of day shows once again how curiously self-centred a large number of medical men manage to keep themselves, even though they presumably live in the midst of a busy world, and how persistently some of them bury their head in the sand and shut their eyes to every view of this question except that which is seen through professional spectacles. Dr. Glover, speaking as one of the direct representatives, maintained that those who opposed all and every kind of legislation which recognised the midwives made the great mistake of being unpractical, and of not taking the actual state of affairs into account. To show what this actual state of affairs really is, he insisted on the

ANNOTATIONS—*continued.*

following three propositions: First, that midwives already exist, and at the present time attend, at a low estimate, 200,000 cases per annum, being recognised and employed not only directly by the poor, but by parish authorities and by maternity charities. Secondly, that the title of midwife is recognised by law, and that nothing in the Medical Act is violated by the title or the practice of the midwife; and, thirdly, that any woman, however drunken, ignorant, or dirty, may without hindrance use this title and under its name may do all sorts of evil. These being the existing conditions, he maintains that Parliament is certain sooner or later to deal with them in some way or another. At present the General Medical Council, which is the only medical body recognised by the Government, is exercising its influence to improve the present Bill. But if by such resolutions as were passed in Manchester it is made plain to all men that the Council does not represent the practitioners, and that the profession as a whole is irreconcilable, then the Government may be driven to settle this question without their co-operation, and to create a perfectly independent class of midwives who shall lay down their own rules and carry out their own discipline independent of the Medical Council and of the medical profession altogether. From long observation of the temper of Parliament in regard to medical affairs we cannot but feel that Dr. Glover's prognostications are correct, and that if medical men persist in regarding this question, not in the light of actual facts, but as if they dwelt in another world, they will awake some fine morning to the discovery that the whole art and mystery of midwifery is entrusted to an absolutely independent body.

### How to Train the Beggar.

In the third annual report of the State Children's Aid Association a fact is brought forward which will astonish many, and shock not a few. It is that on the Derby day the children in the pauper schools at Sutton are marshalled by the roadside, in order that they may receive the coppers that are thrown to them by the people going to the races. The police keep the roadway free from passers-by, so that the children may be seen, and may catch the money thrown to them. The bigger boys run about after the coins that are aimed so ill as to miss the bulk of the children. The infants are placed in a field alongside the railway line, so that as the train slows down they may receive the alms of those who travel by rail. Can one conceive a scheme better devised to make these children, when they grow up, take to begging as a profession? The fact that the money thus received is spent on a day's outing for the children is all the more calculated to make them incline to mendicity as a means of earning a livelihood. Many of these children are the offspring of beggars; their instincts are all in favour of a lazy and facile way of living. But surely it is the duty of those who have the bringing of them up to inculcate upon them in every way the duty of self-reliance, of honest work. To set them begging, even for one day in the year, is bad for the children themselves, and bad for the nation, which wants workers, not beggars, in every sphere. The State Children's Aid Association presented a memorial to Mr. Chaplin, protesting against the practice, but to this they got only a formal acknowledgment, and in answer to a question put in the House of Commons by Mr. Trevelyan, the right honourable gentleman replied that "it amused the children immensely, and so long as he was President of the Local Government Board he would do nothing to interfere with it." It is good to amuse the children, especially the children who spend a dreary and monotonous life in the barrack-schools of a union; but surely amusement can be found of some more wholesome kind than this teaching of the children to beg! Whether, apart from this, the sight of the parties going to and returning from a race-meeting is exactly the spectacle we should like our children to see is another question, serious enough, but not to be compared for a moment with a practice which absolutely teaches children that it is a good and pleasant thing to get money without working for it.

### A Dangerous Weapon.

In days gone by everyone carried a sword; now everyone carries an umbrella, which recent experience shows to be almost as dangerous an instrument. During recent years its construction has been so altered that the harmless gamp, with which at the worst one could but thrash a man, has been turned into a rapier-like instrument, with which it is by no means difficult to run him through, and thus in moments of excitement people find themselves in the possession of a "skewer," the potentialities of which they are hardly aware of. With the object no doubt of giving a slim and dandified appearance, many umbrellas are now made with steel "sticks," and so fine are some of these that the point is very little thicker than the blade of a foil, and is capable of doing quite as much injury if lunged into an antagonist; and this even without the employment of much force, if the proper spot should happen to be entered. Last Saturday a charge of manslaughter was tried at the Central Criminal Court which shows well what may be done with a steel umbrella. As the sequel of a very ordinary quarrel in a public-house, the deceased followed the accused into a room and went up to him, when, as was alleged, the latter thrust an umbrella towards his face. The point entered his cheek, he became unconscious, was taken home in a cab, and died four days afterwards. At the *post-mortem* examination four and a-half inches of the umbrella-stick, which was of iron, were found embedded in his skull, one inch of its length having entered his brain. This piece of iron was stated to have become so firmly fixed that the medical men who performed the *post-mortem* examination had to use a chisel to remove it. The prisoner was acquitted, the jury apparently accepting the statement made by him to the effect that the deceased rushed upon the point of the umbrella, and that the fatal result was accidental. This, however, all the more emphasises what we say about the dangerous character of the modern umbrella with its rapier-like point. If in an ordinary fray, without malice or premeditation, it is possible to bury an umbrella point upwards of four inches deep in a man's head, it is obvious enough that, in the hands of those who are skilled in fence, steel umbrellas must be almost as dangerous as the swords which our great grandfathers used to whip out on the smallest provocation, much to each other's detriment.

